# Effectiveness and safety of digital rectal stimulation and abdominal massage for neurogenic bowel dysfunction in stroke patients: a randomized controlled trial protocol

**DOI:** 10.1186/s13063-023-07678-2

**Published:** 2023-10-03

**Authors:** Sumin Ma, Xiaoyan Fan, Ying He, Chengjuan Li, Dandan Qu, Yanli Man

**Affiliations:** 1 School of Nursing, University of South China, Hengyang, China 421001; 2 The First Hospital of Changsha, Changsha, 410005 China; 3 Department of Rehabilitation, Changsha Central Hospital, Changsha, China 410028; 4 Operating room, Xiangya Hospital, Central South University, Changsha, 410008 China

**Keywords:** Neurogenic bowel dysfunction, Stroke, Rehabilitation

## Abstract

**Background:**

Neurogenic bowel dysfunction (NBD) is a prevalent complication among stroke patients, significantly affecting their quality of life, duration of hospitalization, medical expenses, and even mortality. Although current guidelines suggest a conservative strategy for addressing bowel dysfunction, which includes techniques such as digital rectal stimulation (DRS) and abdominal massage, the availability of interventions remains limited in healthcare facilities.

**Methods:**

This study follows a prospective randomized controlled parallel-group clinical trial design. The control group will receive standard care, while the intervention group will undergo a program that combines DRS and abdominal massage in addition to standard care. The duration of the intervention for both groups will be 6 weeks. The primary outcome measures will be the Wexner score. Furthermore, secondary outcomes measure will be assessed, including Bristol score, Patient Assessment of Constipation-Quality of Life (PAC-QoL), and Fecal Incontinence Quality of Life (FI-QoL).

**Discussion:**

This study aims to evaluate the effectiveness and safety of a bowel rehabilitation program for stroke patients with NBD. The findings will provide information that can contribute to the formulation of bowel management strategies.

**Trial registration:**

The study has been registered in the Chinese Clinical Registry under the number ChiCTR2300071709. This registration was completed on May 23, 2023. All items from the World Health Organization Trial Registration Data set are described in this manuscript.

**Supplementary Information:**

The online version contains supplementary material available at 10.1186/s13063-023-07678-2.

## Background

Lesions in the central nervous system often led to dysregulation of colon function and a lack of coordination in the anal sphincter muscles. Among these lesions, stroke accounts for the highest proportion [1]. When these lesions occur in the brain or brainstem, they can lead to delayed and prolonged transit time of feces through the colon, resulting in constipation [2]. Alternatively, patients may experience increased rectal threshold sensation, abnormal impulse conduction, fecal incontinence, or a combination of these symptoms [2, 3]. Furthermore, stroke patients may already have functional defecation disorders, including acquired dysfunction, prior to disease onset [4]. With the advancement of medicine and the clarification of pathophysiological mechanisms, the Rome Committee is progressively shifting its stance away from the term “functional.” Instead, it considers conditions such as functional constipation, functional diarrhea, or functional bloating as not independent diseases but as having the same underlying mechanisms [5]. Furthermore, Gandel [6] suggested that both functional and secondary bowel functional disorders can benefit from the same treatment strategies.

Bowel dysfunction is prevalent among stroke patients, with a reported incidence of 55.21% post-stroke and 23.96% in pre-stroke patients [7]. Among patients with bowel dysfunction, 50% take more than 30 min on average to defecate, and 70% require assistance during defecation [8]. Additionally, 15% suffer from chronic bowel dysfunction, leading to potential losses in privacy and intimate relationships and social isolation [9]. This places a significant burden of care on the patient’s family [10]. Currently, clinical caregivers heavily rely on empirical information and predominantly employ single intervention, including conservative strategies such as dietary modifications, bowel irrigation medication, or biofeedback training [11–15]. However, certain medications or enemas have the potential to harm the colonic mucosa and decrease the frequency of colonic peristalsis [16]. Surgical treatment is typically reserved for patients without rectal outlet problems who remain unresponsive to intensive medical and biofeedback therapy [13]. Nevertheless, the limited availability of medical equipment might impede patients’ access to biofeedback treatment. Hence, conservative management is recommended for NBD in stroke patients, prioritizing simple, cost-effective, minimally invasive, and reversible interventions [17].

According to guidelines, DRS is recommended as the first-line intervention and has shown a positive impact on improving bowel dysfunction in patients with spinal cord injuries [1, 18]. Moreover, abdominal massage has proven effective in reducing the severity of bowel dysfunction in elderly stroke patients [19]. The combined intervention of abdominal massage and targeted intestinal peristalsis promotion, along with DRS to facilitate fecal elimination, could potentially yield a significantly enhanced effect. However, the concurrent application of these interventions in stroke patients remains infrequent [1]. Robust evidence regarding bowel management is urgently needed. Consequently, this study aims to assess the effectiveness of a 6-week rehabilitation care program that combines DRS and abdominal massage on both bowel function and quality of life in post-stroke patients. We hypothesize that this comprehensive program will be superior to singular care interventions while minimizing complications.

## Methods/design

### Study design

This study is a single-blind, prospective, randomized controlled, parallel-group superiority trial designed to compare the effectiveness of a rehabilitation program that combines DRS with abdominal massage against the standard care provided to stroke patients with NBD.  This trial protocol uses the Standard Protocol Items: Recommendations for Interventional Trials (SPIRIT) reporting guidelines (see Additional file 1). 

### Study setting

The patient data for our study will be collected in Changsha, an area characterized by a high prevalence of stroke and associated mortality [20]. To ensure an adequate participant pool, we will implement a poster recruitment strategy at a level III hospital that houses two rehabilitation departments. These posters will contain information about the study’s objectives, duration, attention matters, and a QR code for registration. Additionally, the contact details of the study coordinator, including a telephone number, will be provided. Interested patients choose to either scan the QR code or contact the study coordinator for further information. This recruitment approach aims to effectively reach potential participants and facilitate their engagement in the study.

### Eligibility criteria

To be eligible for participation in this study, individuals must meet the following criteria: (1) age ≥ 18 years old, (2) stable phase stroke (no stroke relapses within the past 3 months), (3) awake consciousness and stable vital signs, (4) NIHSS score ≤ 15, and (5) bowel dysfunction according to the criteria of Rome IV:

- Constipation is defined as meeting any 2 of the following features:
  - Difficulty with stool passage in 25% or more of bowel movements,
  - Hard stool or lumpy consistency (Bristol Stool Form Scale type 1–2 stool) in 25% or more of bowel movements,
  - Incomplete evacuation in 25% or more of bowel movements,
  - Obstruction or blockage sensation in 25% or more of bowel movements,
  - Need for digital help with 25% or more of bowel movements,
  - Spontaneous bowel movements less than 3 times per week.
- Rare loose stools without the use of laxatives.
- Not meeting the criteria for irritable bowel syndrome.

*The symptoms of constipation must have been present for a minimum of 6 months prior to diagnosis and persisted for the preceding 3 months, in accordance with the aforementioned criteria. It is important to exclude opioid-induced constipation.

Fecal incontinence is defined as recurrent instances of uncontrolled fecal excretion.

*Patients must have experienced symptoms of fecal incontinence for a minimum duration of 6 months prior to diagnosis, with occurrences ranging from 2 to 4 times within the past week.

### Exclusion criteria

The exclusion criteria for patients are as follows: (1) severe cognitive impairment or communication difficulties; (2) history of anorectal trauma and surgery, presence of mass growth in the intestinal canal, or intestinal obstruction; (3) pre-existing condition including Crohn’s disease, inflammatory bowel disease, ulcerative colitis, or irritable bowel syndrome; (4) patients with a confirmed diagnosis of deep vein thrombosis; (5) individuals who are participating in other rehabilitation programs or clinical trials during the study period, and (6) pregnant or lactating women.

## Interventions

### Control group

The participants in the control group will be receive standard care, consisting of dietary guidance, pelvic floor muscle exercises, and skin care. In the event of no enhancement in bowel function over a span of 3 days, prescribed enemas or appropriate medications will be administered as needed.

#### Dietary guidance

Participants experiencing constipation will receive specific instructions to follow a high-fiber diet, with a recommended minimum intake of 15 g per day. However, if a high-fiber intake is not well-tolerated, a lower fiber intake will be advised. Additionally, patients will be encouraged to maintain adequate fluid intake of at least 40 ml/kg, along with an additional 500 ml per day. Moreover, patients will be instructed to schedule defecation approximately 2 h after meals and to develop a regular daily bowel habit.

For participants with fecal incontinence, the dietary recommendations entail a daily consumption of 15 g of dietary fiber. These individuals should also avoid consuming foods and beverages that can result in stool dilution or frequent bowel movements. This list comprises coffee, tobacco, alcohol, fatty foods, fructose, and lactose.

#### Pelvic floor training

The participants will be placed in a supine position, with knees bent and slightly apart. They will receive instructions to contract their anus while inhaling and simultaneously relax their thighs and abdomen muscles. The contraction should be sustained for 3–5 s, followed by a 10-s relaxation period. It is recommended to complete 10–20 repetitions per set, aiming for 4–6 sets daily.

#### Skin care

For participants with a Bristol score of 5 or 6, tampons will be used to manage fecal incontinence. When the Bristol score is 7, stool drainage procedures will be employed. To maintain the skin integrity of patients experiencing fecal incontinence, prompt cleaning of the perineum area will be conducted to ensure cleanliness and dryness. Following the cleaning process, petroleum jelly will be topically applied to the skin, followed by the application of zinc oxide ointment for skin protection.

### Experimental group

Participants in the intervention group will receive an additional intervention that includes DRS and abdominal massage, in addition to the standard care.

### Digital rectal stimulation

The procedure of DRS involves gently inserting either the middle or index finger into the anus. The rectal wall is then gently stimulated in a clockwise direction, creating a circular motion. After completing ten circles, there is a brief pause for 1–2 min. The duration of stimulation is adjusted based on various factors, including the patient’s rectal content, stool consistency, and sphincter muscle strength. The stimulation duration is set at 8 min for mild sphincter injury (anal systolic pressure 15–25 mmHg), 10 min for moderate to severe sphincter injury (anal systolic pressure 0–15 mmHg), and 15 min for severe sphincter injury (anal systolic pressure 0 mmHg). If the patient’s rectal fecal volume exceeds 100 g, the stimulation time is extended accordingly.

### Abdominal massage

**Fig. 1:**
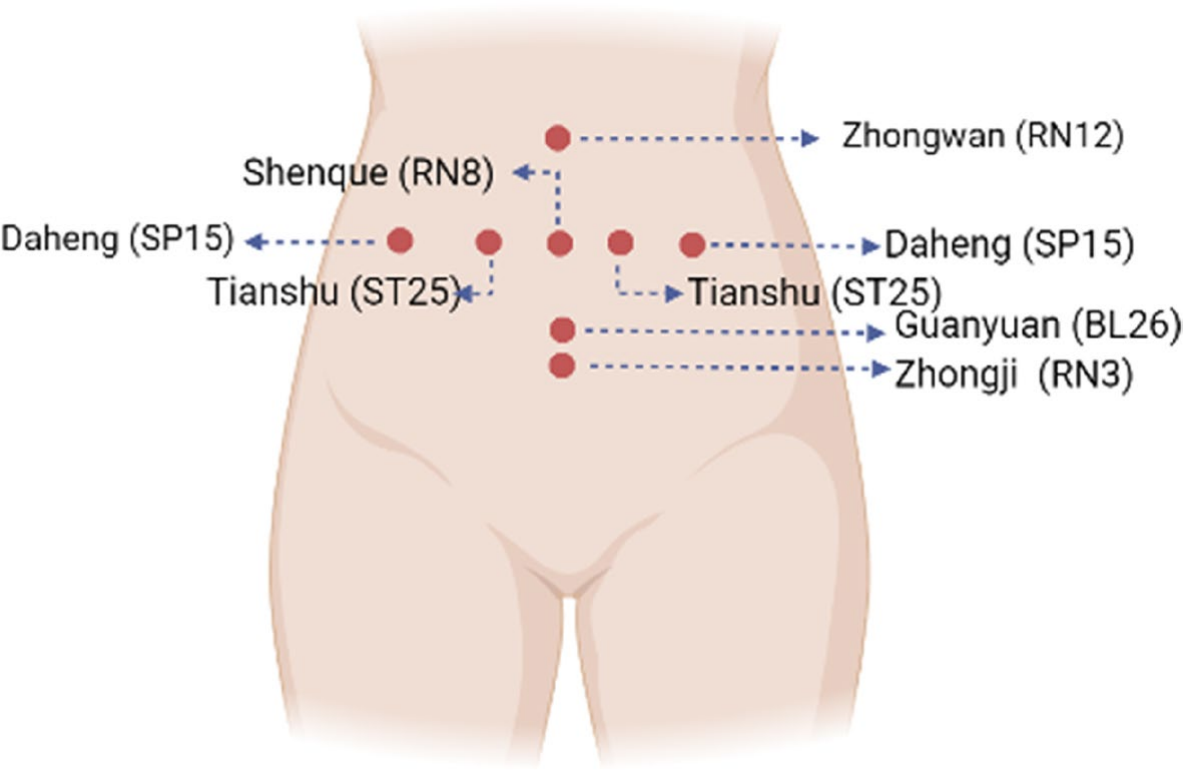
Abdominal massage acupoints

**Fig. 2:**
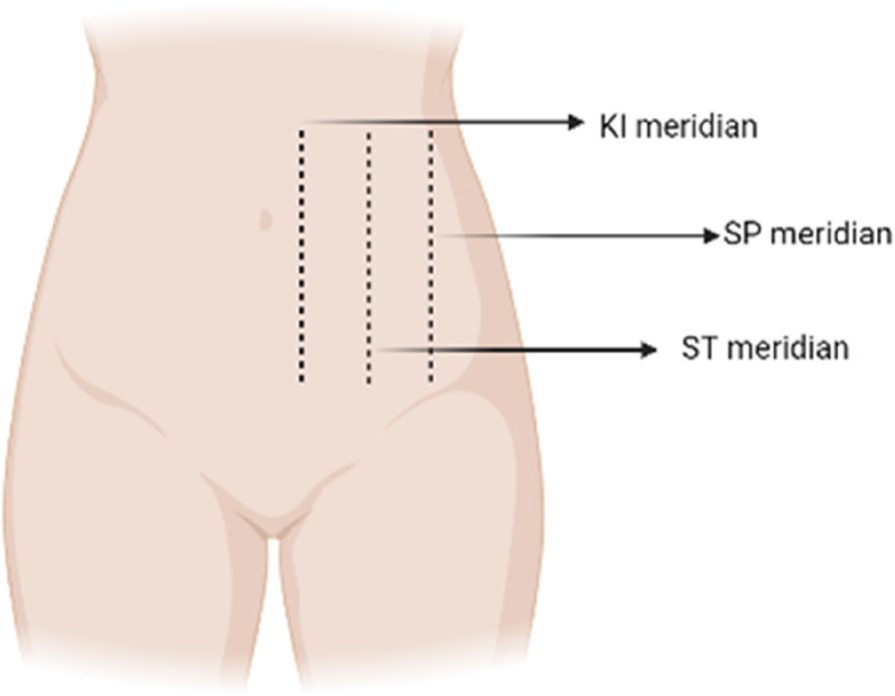
Abdominal massage meridian

- Gently apply warm kneading to the acupoint RN12 (Zhongwan), RN8 (Shenque), BL26 (Guangyuan), RN3 (Zhongji), ST25 (Tianshu), and SP15 (Daheng), rubbing each acupoint for 1 to 2 min. Refer to Fig. 1 for detailed acupoint location.
- Perform warm pushing technique with RN8 (Shenque) as the center. Begin with a small circular motion and gradually increase the circle’s size. Then, reverse the motion from large to small circles. Warmly push the abdomen for 5 min. It is recommended to perform warm pushing from RN12 (Zhongwan) to RN3 (Zhongji), covering the sequence of the kidney meridian, stomach meridian, and spleen meridian. Apply the warm pushing technique to each part 9 to 18 times, using uniform and gentle pressure, while maintaining a slow pace. For detailed acupoint locations, see Fig. 2.
- Repeat the kneading technique on the acupoints RN12 (Zhongwan), RN8 (Shenque), BL26 (Guangyuan), RN3 (Zhongji), ST25 (Tianshu), and SP15 (Daheng). Knead each acupoint for 30 to 60 s.

### Criteria for discontinuing or modifying allocated interventions

Bowel rehabilitation interventions may be discontinued due to the occurrence of adverse effects, exacerbation of health condition, or death from all causes.

### Relevant concomitant care permitted or prohibited

During the 0–6-week period of the study, participants will be instructed to refrain from participating in any other bowel rehabilitation programs. If a participant needs or seeks an alternative form of bowel rehabilitation program during the intervention phase, they will be considered ineligible for participation in the study.

### Provision for post-trial care

Upon completion of the trial, participants who have derived benefits from the intervention and continue to require it will be ensured ongoing access to the intervention. Moreover, if participants in the control group demonstrate a need for the intervention during the study’s duration, they will be offered the chance to receive it.

## Outcomes

### Primary outcome

The primary outcome in this trial is Wexner score, a widely recognized assessment tool extensively employed in clinical practice to assess bowel dysfunction [21]. The Wexner score encompasses both constipation and fecal incontinence scales. The constipation scale comprises 8 items: frequency of defecation, difficulty, completeness, pain assessment, duration of defecation (in minutes), assisted defecation, number of unsuccessful defecations attempts per 24 h, and duration of constipation (in years). The rating of assisted defecation follows a scale of 0–2, while the other items are graded on a scale of 0–4, representing a range from mild to severe [22]. The overall score ranges from 0 to 30, with higher scores indicating greater severity of constipation symptoms.

The Wexner incontinence scale assesses the frequency of gas, liquid, and solid incontinence, in addition to the utilization of pads and lifestyle adjustments [23]. The total score ranges from 0 to 20, with higher scores correlating to increased severity. Item scores vary from 0 to 4, where 0 signifies “never” and 4 signifies “always.” A score of 1 signifies occasional incontinence, while a score of 2 denotes “sometimes,” and a score of 3 indicates “often.”

### Secondary outcomes

#### Bristol Stool Form Scale (BSFS)

Stool consistency will be evaluated using BSFS, a standardized scale that classifies stool consistency into seven categories [24]. Types I and II signify constipation; whereas types III and IV are regarded as the optimal stool shapes, with type IV being the easiest to pass. Types V to VII indicate the potential for diarrhea.

#### Quality of life

The Patient Assessment of Constipation-Quality of Life (PAC-QoL) [25] and the Fecal Incontinence Quality of Life (FI-QoL) instruments are employed to evaluate the quality of life of participants [26]. The PAC-QoL questionnaire consists of four domains: physical discomfort, satisfaction, psychosocial discomfort, and worries and concerns, featuring a total of 28 items. Each item is rated on a 5-point scale (0–4), where higher scores signify a lower quality of life. Meanwhile, the FI-QoL questionnaire is composed of four sections, encompassing a total of 27 questions that assess lifestyle (9 questions), behavior (8 questions), emotion (7 questions), and embarrassment (3 questions). Item scores range from 0 to 4.

#### Sample size

The sample size calculation was performed using the PASS15 software, Version 15.0.5, with reference to a prior randomized controlled trial conducted in China that reported the Wexner score as the primary outcome [27]. We selected a significance level of 0.05 and a power of 90%. Assumptions encompassed an equal allocation of participants in a 1:1 ratio between two groups and a 20% dropout rate. The mean for group 1 (μ_1_) was 10.65, and group 2 (μ_2_) was 9.10. The standard deviation for group 1 (σ_1_) was 2.08, and for group 2 (σ2), it was 1.73. The anticipated effect size was 0.81. The sample size calculation indicated that each group should comprise 40 patients.

## Assignment of interventions

### Allocation—sequence generation

Participants will first be stratified into two groups based on their baseline National Institutes of Health Stroke Scale (NIHSS) scores: one group with scores ranging from 0 to 4 and the other group with scores ranging from 5 to 15. Participants will then randomly be assigned in a 1:1 ratio to either the intervention or control arm. Random numbers within the range of 0 to 1 will be generated using the SPSS 25.0 software by an independent statistician and subsequently ranked. The allocation of participants will be determined based on the ranking of the generated random numbers. For the group with NHISS scores ranging from 0 to 4, odd-ranked numbers will be allocated to the intervention group, while even-ranked numbers will be assigned to the control group. Conversely, in the range of 5 to 15, this allocation will be reversed.

### Allocation—concealment mechanism

The result of random sequences will be placed inside sealed, light-tight envelopes. These envelopes will be prepared by individuals who are not directly involved in the trial. After participants meet the inclusion criteria and provide informed consent, each of them will be given a sealed envelope to open.

## Assignment of interventions: blinding

### Who will be blinded

A third party will oversee the management and coding all data, maintaining the confidentiality of codes until the data analysis is completed. The data analyst will be blinded to the codes. The evaluation of outcomes will be performed by an assessor who is unaware to treatment allocation and has undergone training. However, it is important to note that due to the nature of bowel rehabilitation program, neither implementers of intervention nor participants can be blinded to the intervention.

### Data collection and management

Demographic and baseline information will be collected during the initial assessment. Participants will complete a well-designed questionnaire that encompasses age, sex, height, weight, types of strokes, and medication usage. The data collection will be conducted by a staff member who is not involved in the study. Data will be collected at five time points: during screening, baseline, at post-2-week intervention, at post-6-week intervention, and at 12-week follow-up. The comprehensive data collection plan is presented in Table 1. To ensure confidentiality, each participant will be assigned a unique identifier, and all collected information will be securely stored in an electronic file. Data access will be restricted until statisticians complete the data analysis at the conclusion of the study. After a period of 3 years, all data will be deleted.

**Table 1:**
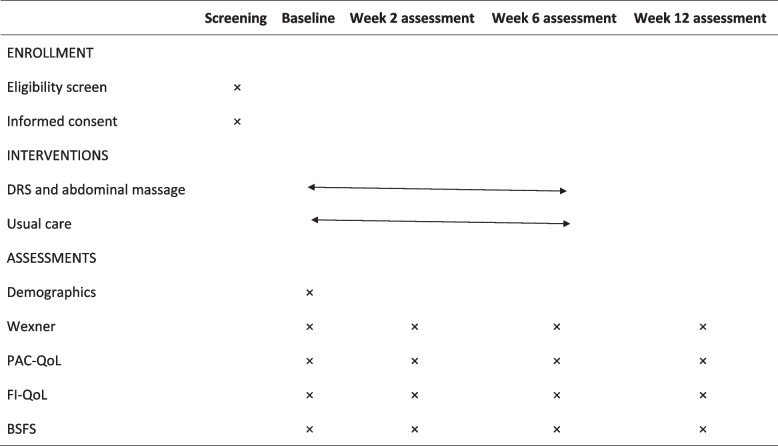
Data collection

### Additional consent provisions for collection and use of participant data and biological specimens

Participants’ stool samples will be collected to assess fecal characteristics using the BSFS. Each participant will be provided with a disposable EasySampler collection kit for stool collection. The collected stool samples will be stored within a temperature range of 10 °C to 25 °C. The analysis of stool samples will involve a visual comparison to the BSFS, allowing for the determination of stool consistency and type. Upon completion of the assessment, typically within 24 h, the stool samples will be promptly and securely destroyed. Informed consent will be obtained prior to collecting these samples. Participants will be informed that any personal information collected during the study will be encoded and kept strict confidentiality. Furthermore, there are no intentions to conduct ancillary studies utilizing the data generated from this trial.

### Plans to promote participant retention and complete follow-up

To facilitate follow-up, participants will receive a bowel intervention diary and on-site observations. To encourage participants’ active involvement in the study, they will be informed the benefits and offered compensation for completing assessments. Regular phone reminders will be sent during the follow-up period.

### Statistical analyses

The collected data will undergo analysis using IBM SPSS Statistics 25.0 (IBM Corp., Armonk, NY, USA). Baseline characteristics will be summarized using descriptive statistics. Continuous variables, such as the Wexner score and quality of life score, will be reported using means, medians, standard deviations, and score ranges. Categorical valuable, such as BSFS, will be reported as counts and percentages. To ascertain normality, the Kolmogorov-Smirnova test will be applied. In instances of normally distributed data, independent samples *t*-test will be employed to compare efficacy between the intervention and control groups in terms of the Wexner score, PAC-QoL, and FI-QoL. Non-normally distributed data will be analyzed using the Wilcoxon rank sum test with a test statistic of *Z*. For assessing changes across distinct time points, we will employ repeated measures ANOVA. The chi-squared test will be used to analyze categorical data for the BSFS. A *p*-value < 0.05 will be considered statistically significant. We will perform an intention to treat analysis for participants who either lack adherence to bowel care (defined as comply with less than 80% of bowel care) or discontinue the study. These participants will be retained within their originally randomized treatment arm for analysis. Addressing missing data, a multiple imputation method will be implemented. No supplementary analyses will be carried out in this study, and the study design does not encompass interim analyses.

### Plans to give access to the full protocol, participant-level data

We will deliver a fully deidentified data set to an appropriate data archive no later than 3 years after the completion of data collection.

### Composition of the coordinating center

The coordinating center team, established at the First Hospital of Changsha, will comprise a multidisciplinary team consisting of a director of nursing, two directors of rehabilitation medicine, and five graduate students. This coordinating center will be tasked roles, including overseeing subject recruitment, personnel management, and implementation hospital-based interventions. The trial steering committee, constituted by members from the School of Nursing, University of South China, will feature seasoned researchers and clinical specialists. The committee members will be responsible for subject recruitment produces, regular monitoring activities, and data verification at the conclusion of the trial.

### Composition and responsibilities of the data monitoring committee

In line with commitment to participant safety and the reliability of research outcomes, an independent data monitoring committee will be instituted. This committee will oversee trial data and adverse reactions as well as provide insightful suggestions. Monthly meetings will be convened to deliberate on trial progress, safety parameters, and data quality. Additionally, meetings will be scheduled in response to any concerns related to participant safety.

### Adverse events and harms

This study identifies adverse events encompassing abdominal pain, anal fissure, and anal bleeding. Rigorous collection of all adverse events, as well as any unexpected reactions, will be executed through both subject self-reports and investigator observations. A standardized classification system in accordance with the Common Terminology Criteria for Adverse Events (CTCAE) will be employed. We will report adverse events categorized as level 2 or higher to the DMC within 24 h. The reporting will include details on severity, occurrence time, duration, actions taken, outcomes, and causality assessment. The follow-up for adverse events will span a period of 12 months post-intervention.

### Frequency and plans for auditing trial conduct

The investigators will provide detailed training videos and booklets to the nurses prior to enrolling the first patients. Regular online meetings will be held weekly to address difficulties in the bowel care process. The Nursing Treatment Control Center of The First Hospital of Changsha will audit the trial every 2 weeks or as necessary.

### Plans for communicating important protocol amendments to relevant parties (e.g., trial participants, ethical committees)

The principal investigator will oversee protocol modifications. Any updated protocols will be promptly reported to the Chinese Clinical Trial Registry, institutional review board (IRB), and all other trial participants and clinical personnel.

### Ethical considerations

This study will adhere to the ethical principles outlined in the Declaration of Helsinki. This study protocol has been approved by the Medical Ethics Committee of the First Hospital of Changsha (number: 215). Informed consent will be obtained from participants or their legal representatives before enrollment. Participants will be informed about the study’s purpose, potential benefits and risks, their rights, and the utilization of anonymized data in scientific publications. The researcher will ensure that participants have a clear understanding of the study. All personal information will be treated with strict confidentiality and privacy. Additionally, participant’s right access information about their medical condition will not be compromised.

### Dissemination plans

The results of this study will be disseminated through diverse channels, with goal of reaching researchers, healthcare professionals, and individuals affected by bowel dysfunction. We intend to achieve this by publishing our findings in international journals, presenting them at academic conferences, and delivering presentations at medical institutions. For future publication authorship eligibility, a minimum trial participation duration of 3 months will be required.

## Discussion

The interventions employed in this study are based on established and effective strategies for NBD. Our investigation is centered on the integration of two interventions: Chinese traditional abdominal massage and DRS, incorporating them into a bowel rehabilitation program for stroke patients. The objective of this intervention is to synergize the strengths of both approaches, aligning with the natural physiological process of defecation [28]. A recent systematic review, which explored neurogenic bowel management among individuals with spinal cord injuries, pointed out that common practice often coupling DRS with complementary therapies like suppositories or enemas [29]. This review highlighted the significance of conducting further research to examine the effectiveness of DRS as a primary intervention. Consequently, DRS will be utilized as a main intervention, along with the innovative integration of traditional Chinese medicine (TCM) message therapy in this study. Drawing upon the principles of TCM, post-stroke patients with NBD often encounter imbalances in yin and yang, disruptions in qi and blood circulation, and the emergence of depressive symptoms. These factors can lead to stagnation of qi and dysfunction of internal organs. The massage therapy of TCM is employed to enhance the defecation reflex and promote intestinal peristalsis. The described interventions offer two advantages: cost-effectiveness and clearly defined parameters that encompass intervention duration and frequency. This approach highlights their inherent potential for replication in various clinical settings.

However, clinical studies in this field lack a standardized definition of bowel dysfunction, and only a limited number of studies utilize the Rome criteria for its definition. This lack of consensus hampers the comparison and interpretation of intervention effectiveness [11, 30]. Additionally, objective indicators are rarely used to assess the effectiveness of interventions, and their impact on patients’ quality of life is often disregarded [7]. In this context, the Wexner system, including both constipation and incontinence scales, will be the primary outcome in this study. Furthermore, neurological injury and quality of life will be evaluated using the NIHSS, PAC-QoL, and FI-QoL, respectively.

In conclusion, the objective of our study is to determine the effectiveness of integrating DRS with TCM abdominal massage as an intervention for managing NBD in stroke patients. We anticipate that our findings will significantly enrich the existing body of evidence related to effective strategies for bowel management.

## Trial status

Registration number: ChiCTR2300071709.

Date when recruitment began: 2023.06.01.

Version identifier: Version 1.0

The date recruitment will be completed approximately: 2023 October 31.

### Supplementary Information

**Additional file 1. **SPIRIT checklist.

## Data Availability

The datasets will be available from the corresponding author upon a reasonable request following to the publication of paper in the journal. Additionally, the complete protocol and materials related to informed consent will also be provided.

## Abbreviations

DRS: Digital Rectal Stimulation
NBD: Neurogenic Bowel Dysfunction
NIHSS: National Institutes of Health Stroke Scale
PAC-QoL: The Patient Assessment of Constipation-Quality of Life
FI-QoL: Fecal Incontinence Quality of Life
TCM: Traditional Chinese Medicine
